# Individualized versus conventional intraoperative blood pressure management among major non-cardiac surgery patients: a systematic review and meta-analysis of randomized controlled trials

**DOI:** 10.3389/fmed.2026.1889492

**Published:** 2026-08-10

**Authors:** Abrar M. Alshammari, Renad Ali, Abdulrahman Mohammed Alkathiry, Abdulrazzaq Qattea, Shahad Algmaizi, Saleh Alminhali, Reem Abdullah Alshehri, Eyad Waleed Halwani, Eibraheim Ahmad S. Al Mrdef, Jihan Jamal Siddiqui, Naif Awwadh Almshrafi, Alzahraa Ahmed Alkhars, Ahmed Elaraby, Ahmad Alkhamis

**Affiliations:** 1 College of Medicine, Alfaisal University, Riyadh, Saudi Arabia; 2 Medical Intern, Ibn Sina National College, Jeddah, Saudi Arabia; 3 GP, General Directorate of Prisons' Health, Al-Ahsa, Saudi Arabia; 4 Department of Emergency Medicine, Al-Quwayiyah Hospital, Al-Quwayiyah, Saudi Arabia; 5 College of Medicine, King Abdulaziz University, Jeddah, Saudi Arabia; 6 College of Medicine, Taif University, Taif, Saudi Arabia; 7 College of Medicine, Al-Rayan National Colleges, Madinah, Saudi Arabia; 8 College of Medicine, Najran University, Najran, Saudi Arabia; 9 International Medical Center, Jeddah, Saudi Arabia; 10 College of Medicine, King Saud Bin Abdulaziz University for Health Sciences, Riyadh, Saudi Arabia; 11 Anesthesia Department, King Faisal General Hospital, Al-Ahsa, Saudi Arabia; 12 Faculty of Medicine, Al-Azhar University, Cairo, Egypt

**Keywords:** acute kidney injury, individualized blood pressure management, intraoperative hypotension, meta-analysis, non-cardiac surgery

## Abstract

**Introduction:**

Intraoperative hypotension is common during non-cardiac surgery and is associated with high rates of morbidity and mortality. Individualized blood pressure management aims to minimize postoperative complications by tailoring patients’ blood pressure targets based on their baseline values rather than relying on conventional management. This meta-analysis aims to evaluate the efficacy of individualized and conventional blood pressure management strategies in patients undergoing non-cardiac surgery.

**Methods:**

We conducted a search of PubMed, Scopus, Web of Science, and Cochrane CENTRAL from their inception to January 2026 to identify randomized controlled trials (RCTs) that compared individualized blood pressure management with conventional care in adults undergoing major non-cardiac surgery. The primary outcomes were acute kidney injury/renal replacement therapy and all-cause mortality. The secondary outcomes included myocardial infarction, delirium, postoperative cognitive dysfunction, composite complications, mean intraoperative mean arterial pressure (MAP), and length of hospital stay.

**Results:**

Nine RCTs comprising 4,017 patients (with 2,014 receiving individualized blood pressure management and 2,003 in the control group) were included. Individualized blood pressure management did not significantly reduce the risk of acute kidney injury/the need for renal replacement therapy (RR 0.88, 95% CI: 0.68–1.15, *p* = 0.36) or all-cause mortality (RR 0.96, 95% CI: 0.63–1.46, *p* = 0.83) compared to conventional management. Similarly, no significant differences were observed for myocardial infarction, postoperative cognitive dysfunction, composite complications, or length of hospital stay. However, individualized management significantly reduced delirium rates (RR 0.55, 95% CI: 0.35–0.88, *p* = 0.01) and achieved higher mean intraoperative MAP (MD 5.53 mmHg, 95% CI: 1.66–9.40, *p* = 0.01).

**Conclusion:**

Individualized intraoperative blood pressure management did not reduce major postoperative complications, such as acute kidney injury, or mortality rates compared to conventional care. However, it was associated with reduced postoperative delirium and improved intraoperative hemodynamic stability. These findings suggest that while individualized strategies may not significantly affect clinical outcomes, they offer benefits in preventing delirium.

**Systematic review registration:**

https://www.crd.york.ac.uk/PROSPERO/view/CRD420261319820.

## 1 Introduction

Intraoperative hypotension is a common complication among patients undergoing non-cardiac surgery, and there is increasing evidence that intraoperative hypotension is associated with an increased risk of postoperative organ injury and death (1). The association between intraoperative hypotension and complications is influenced by both the severity and duration of hypotension (2). A recent systematic review of 42 articles revealed that when intraoperative mean arterial pressure (MAP) drops below 60 mm Hg for 10 min or longer, the risk of acute kidney injury (AKI) increases by 60%, while the risk of myocardial injury rises by 30% (2). However, intraoperative hypotension is a modifiable factor, and interventions such as intravenous fluids and vasopressors can be used to control the MAP.

Guidelines generally recommend maintaining a MAP of at least 60–65 mm Hg during non-cardiac surgery (3); however, the MAP thresholds for adequate organ perfusion vary significantly among individuals (4). Research into individualized or personalized blood pressure management has focused on maintaining intraoperative MAP within a narrow range relative to each patient’s specific preoperative baseline. Early trials suggested potential benefits in reducing morbidity and mortality (5, 6). However, a large-scale randomized controlled trial by Saugel et al. (7) involving patients undergoing abdominal surgery with a high-risk profile for postoperative complications yielded conflicting results.

Therefore, we conducted this systematic review and meta-analysis of RCTs to evaluate the efficacy of individualized intraoperative blood pressure management compared to standard conventional care on postoperative morbidity and mortality in adult patients undergoing major non-cardiac surgery.

## 2 Methods

We conducted this systematic review and meta-analysis following the Preferred Reporting Items for Systematic Reviews and Meta-Analyses (PRISMA) (8) and the Cochrane Handbook of Systematic Review and Meta-Analysis guidelines (9). This study was registered on PROSPERO with ID number: CRD420261319820.

### 2.1 Literature search and screening

We conducted a comprehensive English-language search on PubMed, Scopus, Web of Science (WoS), and Cochrane CENTRAL from their inspection to February 2026 to identify all randomized controlled trials (RCTs) using the following search keywords: (“Intraoperative” OR “Intra-operative” OR “Perioperative” OR “Peri-operative”) AND (“Hypotension” OR “Blood pressure” OR “Arterial pressure” OR “MAP” OR “Mean arterial pressure”) AND (“Individual” OR “Personal”) AND (“Randomized controlled trial” OR “Placebo” OR “Trial” OR “RCT”). Detailed search strategy for each database is summarized in Supplementary Table S1. Following the electronic search, we conducted backward and afterward citation analyses of the reference sections of all retrieved articles to ensure the inclusion of all relevant studies. We first screened the titles and abstracts, followed by a full-text screening to clearly identify eligible studies.

### 2.2 Eligibility criteria and endpoints

We included RCTs that enrolled adults undergoing major non-cardiac surgery, which is defined as non-cardiac operations requiring general anesthesia and major operative/anesthetic exposure, most commonly abdominal, gastrointestinal, urologic, vascular, orthopedic, or other major procedures lasting at least 90–120 min, or involving high-risk surgical patients. Individualized intraoperative BP management was defined as a strategy that establishes intraoperative BP targets based on patient-specific preoperative BP or hemodynamic references, including resting systolic blood pressure (SBP)/MAP, ambulatory or nighttime MAP, or the baseline cardiac index. Conventional intraoperative BP management was defined fas fixed-threshold or usual-care BP treatment, generally consistent with the contemporary practice of avoiding hypotension, with thresholds such as MAP ≥65 mm Hg or SBP ≥80–90 mm Hg (3, 4). Exact target values summarized in Table 1. AKI was identified according to the prespecified criteria established by each trial, which most commonly included Kidney Disease Improving Global Outcomes (KDIGO) stage 1 or higher, RIFLE risk or higher, or a trial-defined rise in postoperative creatinine levels; renal replacement therapy (RRT) was defined as the initiation of postoperative renal replacement therapy. The primary outcomes were the composite renal outcome of AKI or the need for RRT and trial-defined all-cause mortality during the reported postoperative follow-up period. The secondary outcomes were acute myocardial infarction (AMI), delirium, postoperative cognitive dysfunction (POCD), a composite of postoperative complications, mean intraoperative MAP, and length of hospital stay. AMI and myocardial injury were accepted as defined by each trial. Delirium and POCD were assessed using the postoperative assessment tools and timeframes specified by each study. Postoperative composite complications were defined as a composite outcome of infection, pulmonary complications, cardiac complications, kidney complications, gastrointestinal complications, stroke, or death, provided these were part of the original trial’s composite definition.

**Table 1:** Baseline characteristics of the included patients.

Study_ID				Bergholz 2025 (11)	Futier 2017 (5)	Langer 2019 (12)	Lu 2026 (13)	Nicklas 2020 (6)	Nicklas 2024 (14)	Pang 2025 (15)	Saugel 2025 (7)	Zhao 2025 (16)
Sample Size			Intervention	53	147	50	110	94	166	89	567	738
			Control	45	145	51	110	94	162	90	567	739
Age, years		Intervention	Mean	69	69.7	80	71.37	63	66	69	66	68.2
			SD	7.8	7.1	4	6.33	14	10.4	6	4	8.2
		Control	Mean	69	70	80	70.52	63	66	68	66	68.9
			SD	8.1	7.5	4	7.03	14	9.6	5	4	8.2
Gender, Male		Intervention	*N*	21	125	24	66	54	100	58	381	535
			%	40	85	48	60	57.4	60	65.2	66.7	72.5
		Control	*N*	23	123	29	72	60	106	57	366	551
			%	51	85	57	65.4	63.8	65	63.3	64.6	74.6
BMI		Intervention	Mean	75	NR	24.8	23.26	25.3	26.4	22	26.5	23.1
			SD	11.1	NR	4.5	3.2	6.9	5.8	3	3.3	3.7
		Control	Mean	80	NR	24.7	22.6	25.5	26.1	23	26.3	22.6
			SD	10	NR	3.2	2.73	6.2	6.4	3	3.3	3.3
MAP, mmHg		Intervention	Mean	86	NR	NR	NR	94	91	95	84.5	94
			SD	6.7	NR	NR	NR	10	6	10	7.5	10
		Control	Mean	89	NR	NR	NR	94	90	94	82.5	94
			SD	4.1	NR	NR	NR	10	6	10	6.5	10
ASA	Physical status 2	Intervention	*N*	22	62	NR	NR	69	NR	37	235	319
			%	42	42.2	NR	NR	11.7	NR	41.6	41.2	43.3
		Control	*N*	23	54	NR	NR	14	NR	44	235	285
			%	51	37.2	NR	NR	14.9	NR	48.9	41.4	38.6
	Physical status 3	Intervention	*N*	27	84	31	NR	69	NR	52	316	408
			%	51	57.1	62	NR	73.4	NR	58.4	55.3	55.4
		Control	*N*	23	89	33	NR	67	NR	46	316	441
			%	52	61.4	65	NR	71.3	NR	51.1	55.7	1.8
	Physical status 4	Intervention	*N*	4	1	19	NR	14	NR	0	4	9
			%	8	0.7	38	NR	14.9	NR	0	0.7	1.2
		Control	*N*	1	2	17	NR	11	NR	0	7	13
			%	2	1.4	33	NR	11.7	NR	0	1.2	1.8
HTN Medications	Any	Intervention	*N*	45	100	0	110	NR	NR	NR	327	NR
			%	85	68	0	100	NR	NR	NR	57.3	NR
		Control	*N*	38	97	1	110	NR	NR	NR	296	NR
			%	84	66.9	2	100	NR	NR	NR	52.2	NR
	β-Blocker	Intervention	*N*	16	NR	NR	NR	NR	NR	NR	172	78
			%	30	NR	NR	NR	NR	NR	NR	30.1	10.6
		Control	*N*	7	NR	NR	NR	NR	NR	NR	138	92
			%	16	NR	NR	NR	NR	NR	NR	24.3	12.4
	ACEI	Intervention	*N*	13	71	NR	NR	NR	NR	6	117	132
			%	25	48.2	NR	NR	NR	NR	6.7	20.5	17.9
		Control	*N*	3	72	NR	NR	NR	NR	7	101	152
			%	7	49.6	NR	NR	NR	NR	7.8	17.8	20.6
	ARBs	Intervention	*N*	11	71	NR	NR	NR	NR	6	131	132
			%	21	48.2	NR	NR	NR	NR	6.7	22.9	17.9
		Control	*N*	11	72	NR	NR	NR	NR	5	112	152
			%	24	49.6	NR	NR	NR	NR	5.6	19.8	20.6
	CCBs	Intervention	*N*	5	NR	NR	NR	NR	NR	NR	126	265
			%	9	NR	NR	NR	NR	NR	NR	22.1	36
		Control	*N*	7	NR	NR	NR	NR	NR	NR	89	271
			%	16	NR	NR	NR	NR	NR	NR	15.7	36.7
	Diuretics	Intervention	*N*	10	24	NR	NR	NR	NR	NR	111	31
			%	19	16.3	NR	NR	NR	NR	NR	19.4	4.2
		Control	*N*	7	20	NR	NR	NR	NR	NR	87	30
			%	16	13.8	NR	NR	NR	NR	NR	15.3	4.1
Risk Factors	DM	Intervention	*N*	6	77	NR	NR	NR	NR	12	105	210
			%	11	52.4	NR	NR	NR	NR	13.5	18.4	28.5
		Control	*N*	10	73	NR	NR	NR	NR	8	86	222
			%	22	50.3	NR	NR	NR	NR	8.9	15.2	30
	Renal impairment	Intervention	*N*	4	20	6	NR	22	NR	0	396	35
			%	8	13.7	12	NR	23.4	NR	0	69.4	4.8
		Control	*N*	1	17	11	NR	25	NR	0	377	32
			%	2	11.9	22	NR	26.6	NR	0	66.5	4.3
	Expected duration of surgery >2 h	Intervention	*N*	NR	147	18	110	NR	NR	89	448	738
			%	NR	100	36	100	NR	NR	100	78.5	100
		Control	*N*	NR	145	22	110	NR	NR	90	456	739
			%	NR	100	43	100	NR	NR	100	80.4	100
	Smoking	Intervention	*N*	NR	NR	NR	NR	NR	NR	46	133	267
			%	NR	NR	NR	NR	NR	NR	51.7	23.3	36.5
		Control	*N*	NR	NR	NR	NR	NR	NR	40	142	255
			%	NR	NR	NR	NR	NR	NR	44.4	25	34.8
	CAD	Intervention	*N*	4	20	NR	NR	NR	NR	5	71	124
			%	8	13.6	NR	NR	NR	NR	5.6	12.4	16.8
		Control	*N*	5	32	NR	NR	NR	NR	4	67	134
			%	11	22.1	NR	NR	NR	NR	4.4	11.8	18.1
	HF	Intervention	*N*	3	26	NR	NR	NR	NR	6	21	13
			%	8	17.7	NR	NR	NR	NR	6.7	3.7	1.8
		Control	*N*	1	38	NR	NR	NR	NR	6	17	8
			%	2	26.2	NR	NR	NR	NR	6.7	3	1.1
	Cancer surgery	Intervention	*N*	44	NR	28	110	NR	NR	89	430	NR
			%	83	NR	56	100	NR	NR	100	75.3	NR
		Control	*N*	37	NR	34	110	NR	NR	90	427	NR
			%	82	NR	67	100	NR	NR	100	75.3	NR
Surgery type	Abdominal	Intervention	*N*	32	138	30	110	94	166	89	571	738
			%	60	93.9	60	100	100	100	100	100	100
		Control	*N*	28	140	27	110	94	162	90	567	739
			%	62	96.6	53	100	100	100	100	100	100
	Other	Intervention	*N*	21	9	20	0	0	0	0	0	0
			%	40	6.1	40	0	0	0	0	0	0
		Control	*N*	17	5	24	0	0	0	0	0	0
			%	38	3.4	47	0	0	0	0	0	0

*N*, number of patients; SD, standard deviation; BMI, body mass index; MAP, mean arterial pressure; ASA, American Society of Anesthesiologists Physical Status Classification System; HTN, hypertension; ACEI, Angiotensin-Converting Enzyme Inhibitor; ARB, Angiotensin II Receptor Blocker; CCBs, calcium channel blockers; DM, diabetes mellitus; CAD, coronary artery disease; HF, heart failure.

### 2.3 Quality assessment

The risk of bias assessment for RCTs was performed using the Cochrane risk-of-bias tool version 2 (ROB-2) (10). This revised version of the ROB-2 tool assessed five main domains as follows: bias arising from the randomization process, bias arising from deviations from the intended intervention, bias arising from missing outcome data, bias arising from the measurement of outcomes, and bias arising from the selection of reported results. Each domain was classified as having a low risk, some concerns, and a high risk of bias. Any disagreements between the authors were resolved via discussion with the corresponding author.

### 2.4 Data extraction and statistical analysis

We used a standardized Excel sheet to extract all relevant data from the included studies. The extracted data were as follows: (1) summary characteristics of the included studies including: country, study design, study duration, total number of included patients, patients characteristics, the criteria for blood pressure maintenance values for the individualized and control groups, assessed outcomes, and key findings; (2) baseline characteristics of the patients included such as sample size, age, sex (male), BMI, baseline MAP, patients’ comorbidities and risk factors, and surgery type; (3) risk of bias domains; and (4) measured outcomes.

Dichotomous outcomes were extracted as the frequency of events with the total number of patients and were pooled as a risk ratio (RR) with its 95% confidence interval (CI) using the DerSimonian–Laird random effects model. Moreover, continuous outcomes were extracted as the mean, standard deviations (SDs), and the total number of patients, and were pooled as the standardized mean difference (SMD) with its 95% CI using the same model. Significant heterogeneity among the studies was determined when a *p*-value was less than 0.05 and *I*^2^ was ≥50%. A leave-one-out sensitivity analysis was performed to rule out any significant heterogeneity between the studies. STATA 19MP software was used to perform all statistical analyses.

## 3 Results

### 3.1 Literature search and study selection

**Figure 1:**
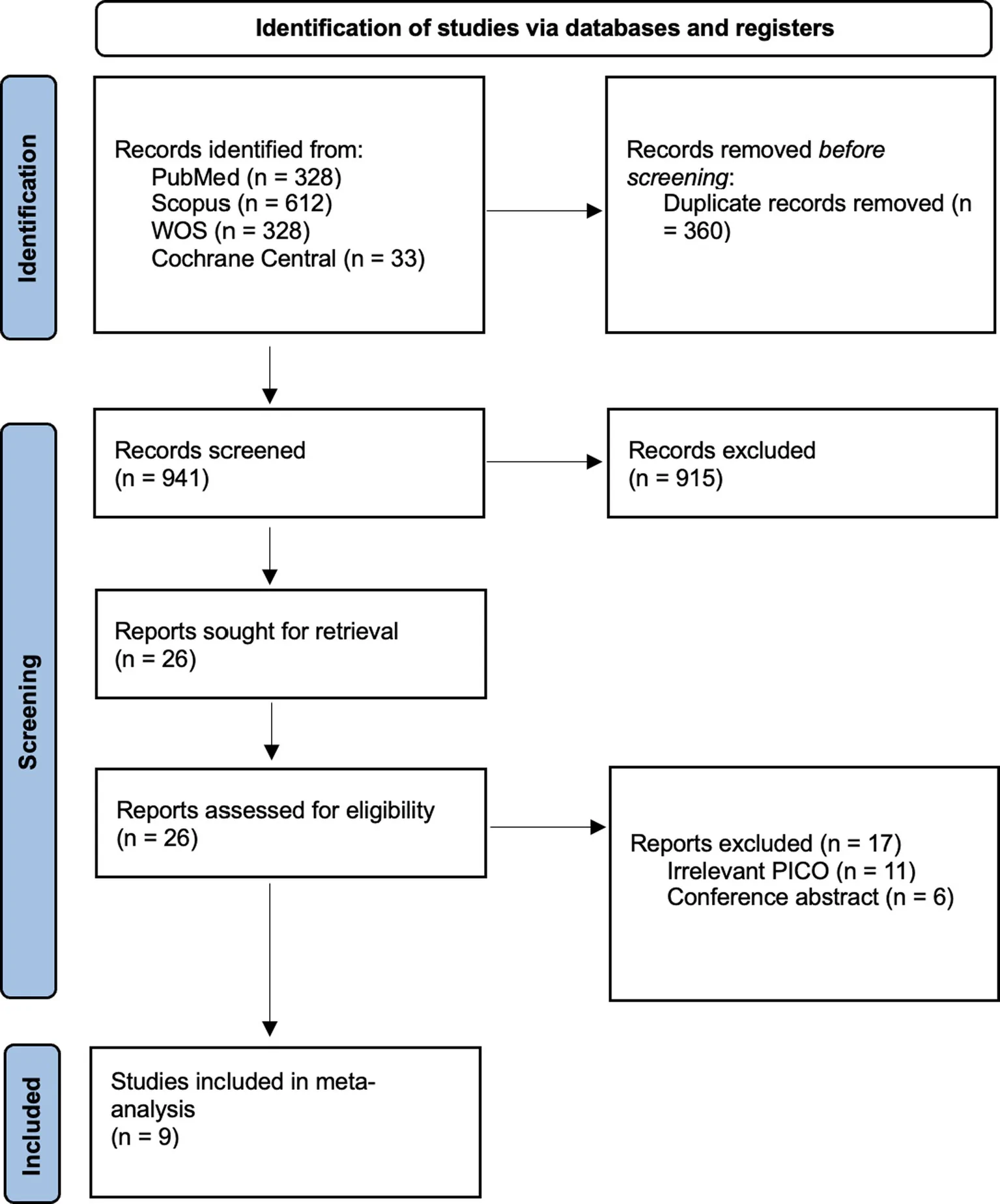
PRISMA flow diagram.

Our comprehensive search yielded 1,301 citations, of which 1,275 were excluded after title and abstract screening and duplicate removal, leaving 26 articles for full-text review. We finally included nine RCTs in the meta-analysis (5–7, 11–16). The selection process is shown in the PRISMA flow diagram (Figure 1).

### 3.2 Study characteristics and quality assessment

The finally included RCTs were conducted in Germany, France, Italy, and China between 2012 and 2024. The studies included a total of 4,017 patients; 68.5% (2,751 patients) were men, with a mean age of 69 ± 7.5 years, a BMI of 31.1 ± 4.9 kg/m^2^, and a MAP of 90.6 ± 8.1 mmHg. The patients were allocated to the individualized group [2,014 (50.1%) patients] and the control group (2,003 (49.9%) patients). More detailed summary characteristics of the included studies and baseline data of the patients are shown in Tables 1, 2.

**Table 2:** Summary characteristics of the included studies.

Study_ID	Country	Study type	Study duration	Total no. patients	Patient characteristics	Surgery type	Intervention	Control	Outcomes assessed	Key findings
Bergholz 2025 (11)	Germany	Bicentric pilot RCT	April 2021–November 2022	98	≥45 years, major non-cardiac surgery, ASA II–IV	Major non-cardiac surgery; abdominal 60–62%; cancer surgery 82–83%	Individualized BP management: maintain MAP > preoperative mean nighttime MAP	Routine BP management: maintain MAP > 65 mmHg	Feasibility of preoperative BP monitoring; proportion with meaningful MAP difference from 65 mmHg; feasibility of maintaining MAP > nighttime MAP; AKI and myocardial injury incidence	93% feasible for BP monitoring; 85% had MAP different from 65 mmHg; maintaining MAP > nighttime MAP feasible; no significant difference in AKI or myocardial injury
Futier 2017 (5)	France	Multicenter RCT	December 2012–August 2016	292	≥50 years, high-risk surgery (AKI risk ≥ III), ASA ≥ II, surgery ≥ 2 h	Predominantly major abdominal surgery; surgery ≥2 h	Individualized SBP within ±10% of preoperative resting value (norepinephrine infusion)	Standard SBP < 80 mmHg or <40% from baseline (ephedrine bolus)	Composite of SIRS + organ dysfunction by day 7; individual organ dysfunctions; 30-day mortality	Lower composite organ dysfunction (38.1% vs. 51.7%); lower renal dysfunction and altered consciousness; no difference in mortality
Langer 2019 (12)	Italy	Single-center pilot RCT	November 2014–April 2016	101	≥75 years, elective non-cardiac surgery, ASA < 4	Elective non-cardiac surgery under general anesthesia; abdominal about 53–60%	MAP ≥ 90% of preoperative value (Target group)	Liberal BP management (non-target group)	POCD at 3 months; delirium; feasibility outcomes (time at target, recruitment/dropout)	Higher time at target in intervention group (65% vs. 49%); no difference in POCD or delirium; recruitment 6/month; dropout 24%
Lu 2026 (13)	China	Multicenter RCT	January 2019–December 2021	220	65–80 years, hypertension, major gastrointestinal surgery, ASA I–III	Major gastrointestinal surgery; all cancer surgery	Individualized BP: SBP ± 10% or MAP ±20% of baseline (norepinephrine infusion)	Conventional BP: MAP 80–95 mmHg	Serum creatinine change; microRNA-21-5p levels; AKI incidence; postoperative complications	Lower Cr at 7 days; lower microRNA-21-5p perioperatively; no difference in AKI; higher vasopressor use in the intervention group
Nicklas 2020 (6)	Germany	Single-center RCT	May 2016–June 2017	188	Adults ≥18 years, major abdominal surgery ≥90 min, at least one high-risk criterion	Major abdominal surgery ≥90 min	Individualized hemodynamic management: Maintain intraoperative CI at individual preoperative baseline CI (measured at rest preoperatively) using fluids/dobutamine algorithm	Routine management (anesthesiologist preference, typically MAP ≥65 mmHg)	Composite of major complications or death within 30 days, POMS, hospital LOS, 90-day mortality, neurocognitive function, systemic inflammation	Significant reduction in major complications. Mortality: 1 vs. 5 deaths. Improved neurocognitive function in some tests
Nicklas 2024 (14)	Germany	Single-center RCT	March 2018–October 2019	328	≥50 years, Elective major noncardiac surgery ≥90 min, ASA 2–4	Elective major non-cardiac surgery ≥90 min; all abdominal in extracted baseline table	Individualized BP management: maintain intraoperative MAP above individual preoperative baseline MAP (from 24-h ambulatory BP monitoring)	Routine management (MAP ≥65 mmHg)	Incidence of neurocognitive disorders (composite of delayed neurocognitive recovery/delirium) within postoperative days 3–7, AKI, myocardial injury, complications, mortality, LOS	No significant difference in neurocognitive disorders. Delayed neurocognitive recovery: 12% vs. 12%. Delirium: 1% vs. 3%
Pang 2025 (15)	China	Single-center Pilot RCT	March 2022–June 2023	179	≥60 years, Elective major abdominal surgery, ASA I-III	Elective major abdominal surgery; laparoscopic gastric/intestinal tumor resection	Individualized BP management: target SBP within −20 to +10% of baseline (if baseline ≥130/80 mmHg), otherwise within ±10% of baseline	Routine management (SBP ≥ 90 mmHg and MAP ≥65 mmHg)	Incidence of AKI within 7 postoperative days (KDIGO criteria), intraoperative hypotension, urine output, mechanical ventilation, delirium, complications, mortality	Non-significant 28% reduction in AKI. Markedly reduced hypotension (3% vs. 89% with MAP<65 mmHg). More urine output, shorter mechanical ventilation, faster bowel recovery
Saugel 2025 (7)	Germany	Multi-center RCT	February 2023–April 2024	1,134	≥45 years, major abdominal surgery ≥90 min, at least one high-risk criterion	Major abdominal surgery ≥90 min; all abdominal; cancer surgery about 75%	Individualized BP management: Maintain MAP ≥ individual preoperative mean nighttime MAP (from ambulatory monitoring), min 65 mmHg, max 110 mmHg	Routine management (MAP ≥65 mmHg)	Composite of AKI, acute myocardial injury, nonfatal cardiac arrest, or death within 7 days, 22 outcomes including infectious complications; composite of RRT/MI/arrest/death at 30/90 days	No significant difference in the composite outcome. All 22 secondary outcomes were non-significant. Individualized group received more norepinephrine
Zhao 2025 (16)	China	Multicenter Investigator-initiated, parallel-group RCT	30 June 2020–23 September 2022	1,477	≥45 years, Known cardiovascular disease or risk factors, Scheduled for inpatient major abdominal surgery ≥2 h	Inpatient major abdominal surgery expected to last ≥2 h; all abdominal	Conventional intraoperative BP management: Target MAP ≥ the higher of 65 mm Hg or 60% of preoperative baseline	Intensive intraoperative BP management: Target MAP ≥80 mmHg	Composite of cardiovascular complications within 30 days (myocardial injury/infarction, new-onset arrhythmias, acute heart failure, stroke, cardiac arrest, all-cause death). Non-cardiovascular complications within 30 days, days alive and at home within 30 days, and death or new disability after 180 days.	Intensive management successfully reduced hypotension exposure. No significant differences in secondary outcomes.

RCT; randomized controlled trial, ASA; American Society of Anesthesiologists Physical Status Classification System, BP; blood pressure, MAP; mean arterial pressure, SBP; systolic blood pressure, AKI; acute kidney injury, SIRS; systemic inflammatory response syndrome, POCD; postoperative cognitive dysfunction, POMS; Postoperative Morbidity Survey, LOS; length of stay, KDIGO; Kidney Disease: Improving Global Outcomes, RRT; renal replacement therapy, MI; myocardial infarction, Cr; creatinine.

**Figure 2:**
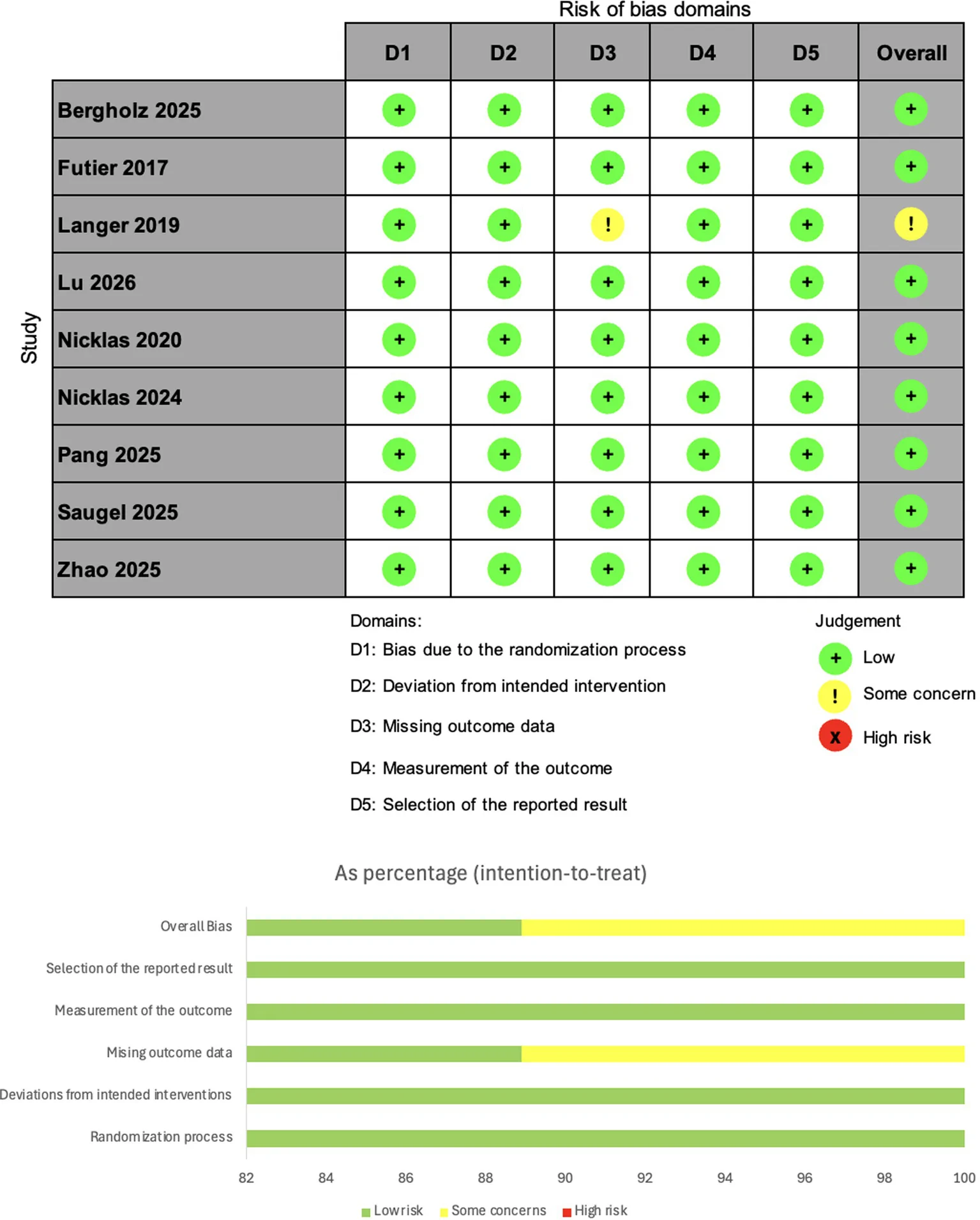
ROB assessment (ROB-2) of the included RCTs.

As shown in Figure 2, the nine included RCTs were assessed using the ROB-2 tool, and eight studies had a low risk of bias, while only one study had some concern for bias due to missing outcome data (12).

### 3.3 Primary outcomes

**Figure 3:**
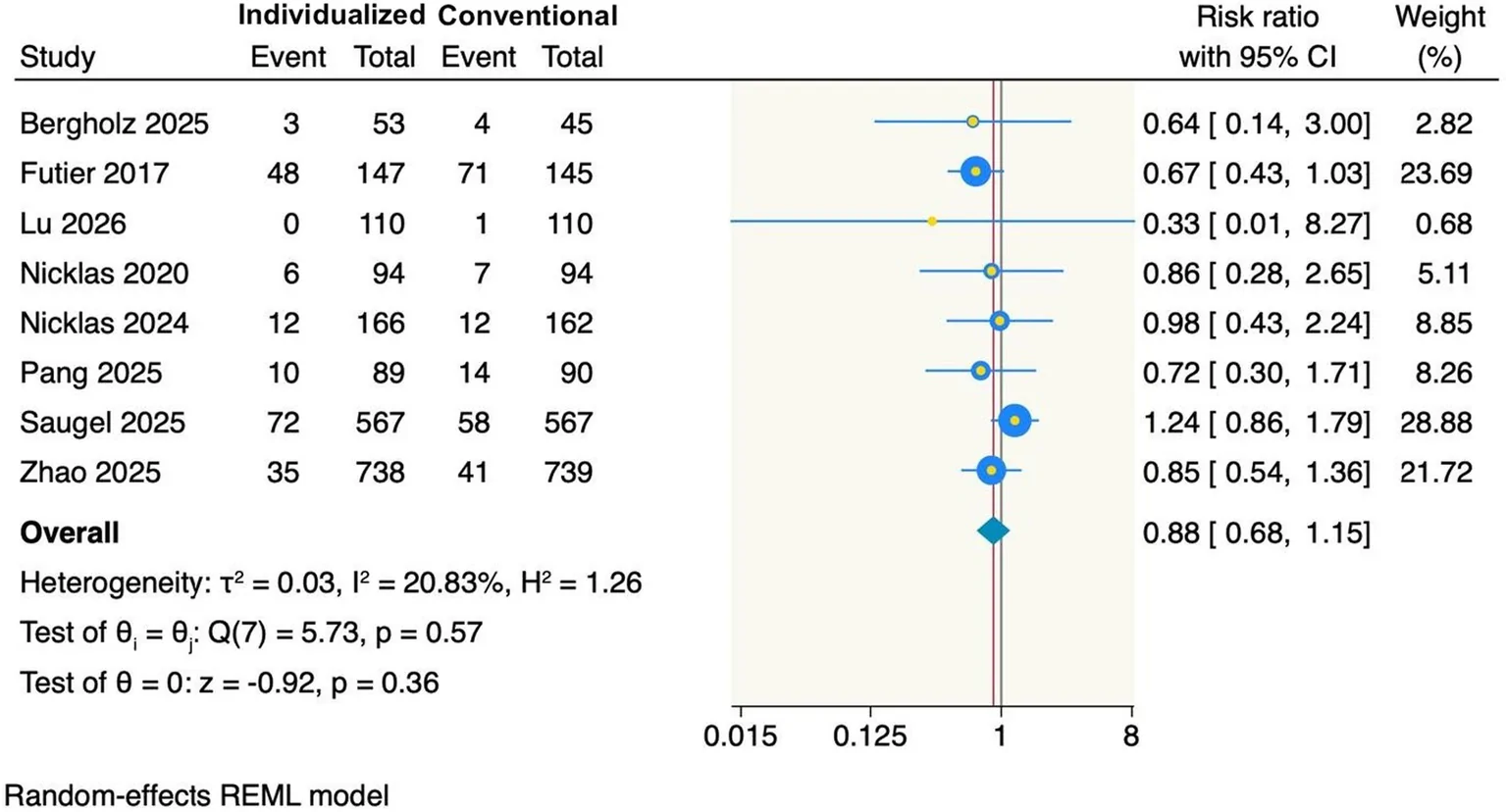
Random-effect model of AKI/RRT.

The incidence of AKI/the need for RRT was assessed in eight studies, with rates of 9.47% (186 of 1,964 patients) in the individualized group and 10.65% (208 of 1,952 patients) in the control group. The pooled analysis showed no significant difference between individualized and conventional BP strategies to reduce the risk of AKI/RRT (RR 0.88, 95% CI: 0.68 to 1.15, *p* = 0.36; *I*^2^ = 20.83%, *p* = 0.57; Figure 3).

**Figure 4:**
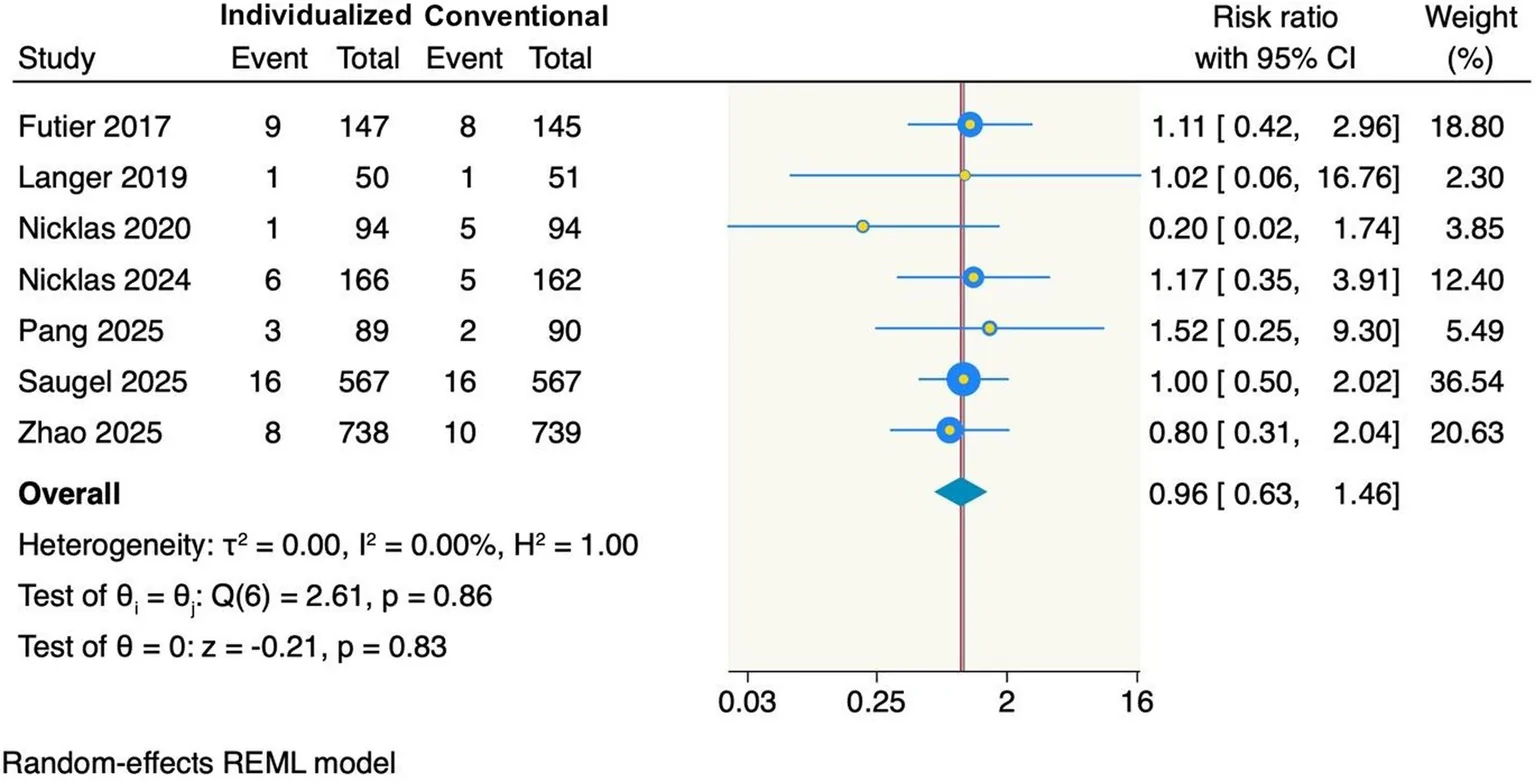
Random-effect model of all-cause mortality.

The incidence of all-cause mortality was reported in seven studies; the rates were similar between the two groups: 2.37% (44 of 1,851 patients) in the individualized group and 2.54% (47 of 1,848 patients) in the control group. The pooled estimate showed no significant difference between the two strategies to reduce mortality rates (RR 0.96, 95% CI: 0.63 to 1.46, *p* = 0.83; *I*^2^ = 0%, *p* = 0.86; Figure 4).

### 3.4 Secondary outcomes

As shown in Supplementary Appendix, five studies reported the MI incidence among patients, and the pooled analysis showed no significant difference between the individualized and control groups (RR 1.03, 95% CI: 0.81 to 1.32, *p* = 0.78; *I*^2^ = 0%, *p* = 0.30). Five studies reported delirium rates, and the pooled estimate showed a significant reduction in delirium rates between the individualized and control groups (RR 0.55, 95% CI: 0.35 to 0.88, *p* = 0.01; *I*^2^ = 17.33%, *p* = 0.54). Only three studies reported postoperative cognitive dysfunction rates, and the pooled analysis showed no significant difference between the two studied groups (RR 0.74, 95% CI: 0.44 to 1.26, *p* = 0.27; *I*^2^ = 28.11%, *p* = 0.29). Moreover, five studies reported the incidence of postoperative composite complications, and the pooled estimate showed no significant difference in complication rates between the individualized and control groups (RR 0.91, 95% CI: 0.77 to 1.08, *p* = 0.27; *I*^2^ = 0%, *p* = 0.87; Table 3).

**Table 3:** Methods of BP personalization.

Study_ID	Intervention target	Intervention technique	Control target	Control technique
Bergholz 2025 (11)	Maintain intraoperative MAP ≥ preoperative mean nighttime MAP (max target 100 mmHg). If nighttime MAP < 65 mmHg, target ≥ 65 mmHg.	No specific hemodynamic protocol; clinicians used their discretion to maintain the target MAP.	Maintain MAP > 65 mmHg.	Institutional routine; clinicians were blinded to nighttime MAP and used their discretion.
Futier 2017 (5)	SBP within ±10% of the patient’s resting reference value.	Continuous intravenous infusion of norepinephrine adjusted according to a dedicated table.	SBP < 80 mmHg or lower than 40% from the reference value.	Intravenous ephedrine in 6-mg boluses (max 60 mg). Norepinephrine permitted as rescue therapy.
Langer 2019 (12)	MAP ≥ 90% of preoperative baseline MAP.	Strategy to reach the hemodynamic target was at the discretion of the anesthesiologist.	Liberal intraoperative blood pressure management (no target specified).	Usual practice without specified targets; strategy at the discretion of the anesthesiologist.
Lu 2026 (13)	SBP maintained at ±10% or MAP maintained at ±20% of the baseline level.	Continuous noradrenaline administration. If MAP deviated, boluses of phenylephrine, ephedrine, nitroglycerin, or phentolamine were used to return to target within 5 min.	MAP maintained at 80–95 mmHg.	Not explicitly tied to specific vasopressor protocol in text; moderately liberal fluid regimen used for both groups.
Nicklas 2020 (6)	Maintain intraoperative Cardiac Index at individualized preoperative values measured at rest.	Specific treatment algorithm using 500 mL fluid boluses and dobutamine. Norepinephrine used to keep MAP 65–90 mmHg.	Maintain MAP > 65 mmHg and maintain normovolemia.	Anaesthesiologist preference using crystalloid/colloid fluids. Norepinephrine as first-line vasopressor.
Nicklas 2024 (14)	MAP above preoperative baseline MAP (ideally baseline MAP + 10 mmHg; min range 65–75 mmHg, max range 100–110 mmHg).	No specific hemodynamic management protocol; clinicians used their discretion to maintain target MAP.	Maintain MAP > 65 mmHg.	Managed per institutional routine at clinicians’ discretion; unaware of automated baseline data.
Pang 2025 (15)	If baseline SBP ≥ 130 or DBP ≥ 80: SBP target between 80 and 110% of baseline (max 160). Otherwise: SBP within ±10% of baseline and MAP 65–95 mmHg.	Metaraminol infusion adjusted per protocol. Goal-directed fluid management used in both arms.	SBP ≥ 90 mmHg and MAP ≥ 65 mmHg.	Metaraminol (0.2–0.5 mg) administered as necessary to sustain targets.
Saugel 2025 (7)	MAP ≥ preoperative mean nighttime MAP. (If < 65, target ≥ 65 mmHg; if > 110, target ≥ 110 mmHg).	Interventions were determined by treating anesthesiologists; no specific hemodynamic protocol.	Maintain MAP ≥ 65 mmHg.	Interventions were determined by treating anesthesiologists; no specific hemodynamic protocol.
Zhao 2025 (16)	MAP ≥ 80 mmHg.	Continuous infusion of norepinephrine starting at 0.01 μg/kg/min and adjusted as necessary.	MAP ≥ the higher of 65 mmHg or 60% of the patient’s preoperative baseline.	Continuous infusion of norepinephrine starting at 0.01 μg/kg/min and adjusted as necessary.

**Figure 5:**
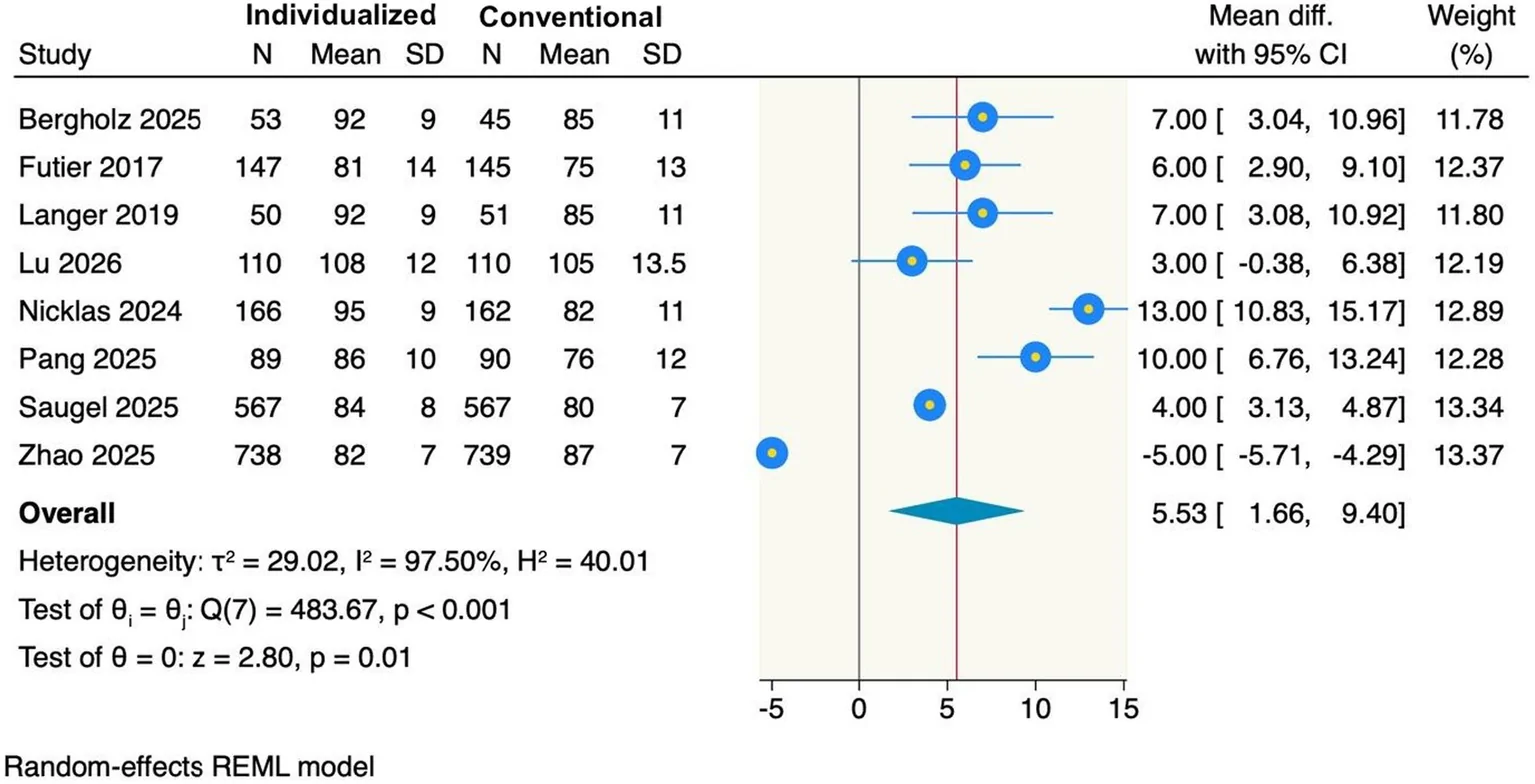
Random-effect model of mean intraoperative MAP.

The mean intraoperative MAP was assessed in eight studies, and the pooled estimate showed a significantly higher MAP of 5.53 mmHg in the individualized group (MD 5.53, 95% CI: 1.66 to 9.40, *p* = 0.01; *I*^2^ = 97.50%, *p* < 0.001; Figure 5). We performed leave-one-out sensitivity analysis, and by inspection, no single study had a disproportional effect over the pooled MD (Supplementary Appendix). The length of hospital stay was reported in seven studies, and the pooled estimate showed no significant difference between the two studied groups (MD -0.13, 95% CI: −0.97 to 0.70, *p* = 0.75; *I*^2^ = 76.38%, *p* < 0.001). The leave-one-out analysis showed that no single study affected the pooled MD (Supplementary Appendix).

## 4 Discussion

Our meta-analysis, which includes 9 randomized controlled trials conducted across multiple countries with a total number of 4,017 patients undergoing major non-cardiac surgery, provides the most comprehensive evaluation of individualized intraoperative blood pressure management compared to the conventional management strategy.

Our pooled analysis showed no significant difference between individualized and conventional BP management strategies to reduce the risk of acute kidney injury or renal replacement therapy (AKI/RRT) or the incidence of all-cause mortality. However, among the secondary outcomes, individualized BP strategy showed a significant reduction of 45% in postoperative delirium rates compared to the conventional strategy. However, there were no significant differences between the two strategies regarding the incidence of myocardial infarction (MI), postoperative cognitive dysfunction (POCD), postoperative composite complications, or the overall length of hospital stay. Finally, the intraoperative MAP was significantly higher by 5.53 in the individualized BP management groups compared to the conventional management groups.

The number of major non-cardiac surgeries is growing globally; however, hemodynamic fluctuation, particularly hypotension during the surgery, is considered a risk factor for kidney, brain, and heart injury (17). Recently, an individualized BP management strategy was proposed as an alternative to conventional management, with conflicting results between the trials. The multi-center INPRESS trial (5) was conducted on 292 patients undergoing abdominal surgery and compared the individualized blood pressure management strategy aimed at achieving a systolic blood pressure (SBP) within 10% of each patient’s baseline value versus a routine management strategy of SBP less than 80 mm Hg or lower than 40% from the baseline value. The trial reported that the individualized BP management strategy was associated with a significant reduction in the incidence of systemic inflammatory responses and composite outcomes related to organ failure (5). Furthermore, they reported a significant reduction in renal dysfunction (RIFLE stage of risk or higher) in the individualized treatment group compared to the standard treatment group, with an absolute risk difference of −16% (5). However, their results were not aligned with our pooled estimate; although there was a significant pooled increase of 5.53 mmHg in the intraoperative MAP associated with individualized treatment groups compared to the standard treatment groups, this was not translated into an overall benefit in the postoperative clinical outcomes. The neutral effect on major complications may also reflect effective prevention of hypotension in the conventional arms. Several trials achieved only modest separation in achieved intraoperative BP, and contemporary conventional care commonly targeted a MAP of ≥65 mm Hg. In cases where the differences were clear, such as in the study by Zhao et al. (16), which found that the intensive arm had less time below a MAP 65 of mm Hg, and in the study by Pang et al. (15), where the individualized arm had markedly lower hypotension exposure, there was still no statistically significant reduction in major renal or cardiovascular outcomes. Therefore, the comparison may be less about individualized targets versus unprotected hypotension and more about comparing higher or baseline-derived targets to reasonably effective conventional methods for avoiding hypotension. This aligns with the recent IMPROVE trial; the IMPROVE-multi trial randomized 1,142 high-risk major abdominal surgery patients from 15 German hospitals to individualized perioperative BP management—targeting a MAP at or above the preoperative mean nighttime MAP, with protocol boundaries set between 65 and 110 mm Hg—or conventional management, targeting a MAP of ≥65 mm Hg (7). The trial reported no significant difference in the primary composite outcome of acute kidney injury, acute myocardial injury, non-fatal cardiac arrest, or death within the first 7 postoperative days, nor in any of the 22 secondary outcomes (7). Pang et al. conducted a trial on 192 patients and reported that individualized management was not associated with a reduction in the incidence of AKI (15).

Taken together, these trial-level findings are consistent with prior evidence syntheses. Our findings should be interpreted alongside prior evidence on intraoperative hypotension and BP optimization. Wesselink et al. reported that lower intraoperative MAP thresholds and longer hypotension duration were associated with postoperative organ injury, especially AKI and myocardial injury (2). A meta-analysis of randomized trials comparing permissive and targeted intraoperative BP management found uncertain effects on major postoperative outcomes (18). A systematic review of BP optimization strategies in major non-cardiac surgery concluded that many interventions reduce hypotension exposure but do not consistently improve patient-centered outcomes (19). A recent higher-versus-routine BP target meta-analysis similarly suggested that higher targets may reduce hypotension exposure without clearly reducing AKI, mortality, or cardiovascular events (20). Our RCT-only synthesis, which focuses on individualized versus conventional strategies, is consistent with this broader pattern: better intraoperative BP separation does not necessarily translate into broader postoperative benefits; however, it appears that delirium may be more sensitive to individualized targets.

Intraoperative management strategies have been under investigation in the last decade; however, our pooled evidence demonstrates that individualized treatment strategies do not offer a significant benefit in terms of clinical outcomes. This might be attributed to the fact that individualized treatment with higher MAP targets often require more vasoactive support. Norepinephrine/noradrenaline was the dominant vasopressor in the larger BP-target trials, including those by Futier, Bergholz, Nicklas (2024), Saugel, Zhao, and Lu. Other agents included ephedrine, phenylephrine, metaraminol, and dobutamine in select protocols. (21). This may create a discordance between higher measured arterial pressure and effective organ perfusion: vasopressors increase macrovascular pressure, but excessive vasoconstriction may reduce microcirculatory blood flow, particularly in renal and splanchnic beds. This mechanism could blunt renal benefit and may contribute to organ dysfunction despite higher MAP. In addition, trials differed in how they defined the individualized baseline reference. The IMPROVE (7) trial and Nicklas et al. (14) used ambulatory or nighttime baseline MAP, which may not fully represent intraoperative autoregulatory thresholds that vary across patients and over time (22). Other trials used different references: Futier et al. (5) used preoperative resting SBP, Langer et al. (12) used preoperative MAP, Lu et al. (13) used baseline SBP or MAP, Pang et al. (15) used baseline SBP/DBP to set an individualized SBP range, and Nicklas et al. (6) targeted the baseline cardiac index rather than BP alone.

A significant 45% relative risk reduction in postoperative delirium rates was observed in the individualized BP management groups, pointing to the sensitivity of the aging brain to intraoperative hypotension. Patients undergoing major surgery often present with chronic hypertension leading to vascular remodeling, endothelial hypertrophy, and impaired autoregulation (21). While a MAP of 65 mm Hg is traditionally considered safe, it might be low for cerebral autoregulation in chronically hypertensive patients. This could induce silent cerebral micro-ischemia and subsequent delirium (14).

### 4.1 Clinical implications

The individualized BP management strategy achieves a higher intraoperative MAP; however, this optimization does not yield clinical benefits for cardiovascular or renal outcomes. However, the significant reduction in delirium rates suggests a neuroprotective advantage of individualizing intraoperative blood pressure targets.

### 4.2 Limitations

While this systematic review and meta-analysis provides the most up-to-date synthesis of individualized hemodynamic management strategies compared with conventional management, it has several limitations that should be considered when interpreting the results. First, BP was the primary focus of this review. Other hemodynamic variables, including cardiac output/index, stroke volume, dynamic preload responsiveness, and preload itself, were not pooled. In addition, BP measurement method and frequency, intravascular fluid status, surgical stress, anesthetic agents, myocardial depressant drugs, and perioperative antihypertensive management varied across trials. These factors may modify the relation between measured arterial pressure and true organ perfusion. The second limitation is unblinded intraoperative management due to the nature of the intervention; anesthesiologists titrating the vasopressors could not be blinded to the assigned individualized blood pressure targets, introducing a potential performance bias. Finally, most included trials enrolled high-risk adults undergoing major abdominal or other major non-cardiac surgery; intermediate-risk operations were not represented. Therefore, these findings should not be generalized to lower-risk or intermediate-risk procedures without caution.

## 5 Conclusion

Individualized intraoperative BP management successfully maintains a higher intraoperative MAP and provides clinical benefits, including postoperative delirium among patients undergoing major non-cardiac surgery. However, it does not reduce the incidence of acute kidney injury, major cardiovascular events, or overall mortality.

## Data Availability

The original contributions presented in the study are included in the article/Supplementary material, further inquiries can be directed to the corresponding author.
