# Supplementary material for: Individualized versus conventional intraoperative blood pressure management among major non-cardiac surgery patients: a systematic review and meta-analysis of randomized controlled trials
Source: Front Med (Lausanne). 2026 Aug 10;13:1889492. doi: 10.3389/fmed.2026.1889492 (PMC13500609; doi:10.3389/fmed.2026.1889492)
Supplement: Supplementary file 1 [file Supplementary_file_1.docx]

**Supplementary Appendix**

| ***Content*** | ***Page*** |
| --- | --- |
| **Supplementary Table S1.** Detailed search strategy for each database. | **2** |
| **Supplementary Table S2.** The detailed baseline characteristics |  |
| **Supplementary Figure S1.** Random-effect model of MI rates. | **3** |
| **Supplementary Figure S2.** Random-effect model of delirium rates. | **4** |
| **Supplementary Figure S3.** Random-effect model of postoperative cognitive dysfunction rates. | **5** |
| **Supplementary Figure S4.** Random-effect model of postoperative composite complications rates. | **6** |
| **Supplementary Figure S5.** Random-effect model of length of hospital stay. | **7** |
| **Supplementary Figure S6.** Leave-one-out sensitivity analysis of mean intraoperative MAP. | **8** |
| **Supplementary Figure S7.** Leave-one-out sensitivity analysis of length of hospital stay. | **9** |

**Supplementary Table S1.** Detailed search strategy for each database.

| Database | Search Term | Filter | Date | Results |
| --- | --- | --- | --- | --- |
| PubMed | ("Intraoperative" OR "Intra-operative" OR "Perioperative" OR "Peri-operative") AND ("Hypotension" OR "Blood pressure" OR "Arterial pressure" OR "MAP" OR "Mean arterial pressure") AND ("Individuali*" OR "Personali*" OR "Optimis*" OR "Optimiz*" OR "Tailor*") AND ("Randomized controlled trial" OR "Randomized" OR "Randomised" OR "Placebo" OR "Trial" OR "RCT") | English, All articles | 19 Jan 2026 | 328 |
| Scopus | ("Intraoperative" OR "Intra-operative" OR "Perioperative" OR "Peri-operative") AND ("Hypotension" OR "Blood pressure" OR "Arterial pressure" OR "MAP" OR "Mean arterial pressure") AND ("Individuali*" OR "Personali*" OR "Optimis*" OR "Optimiz*" OR "Tailor*") AND ("Randomized controlled trial" OR "Randomized" OR "Randomised" OR "Placebo" OR "Trial" OR "RCT") | English, Article title, abstract, keywords | 19 Jan 2026 | 612 |
| WOS | ("Intraoperative" OR "Intra-operative" OR "Perioperative" OR "Peri-operative") AND ("Hypotension" OR "Blood pressure" OR "Arterial pressure" OR "MAP" OR "Mean arterial pressure") AND ("Individuali*" OR "Personali*" OR "Optimis*" OR "Optimiz*" OR "Tailor*") AND ("Randomized controlled trial" OR "Randomized" OR "Randomised" OR "Placebo" OR "Trial" OR "RCT") | English, All articles | 19 Jan 2026 | 328 |
| Cochrane | ("Intraoperative" OR "Intra-operative" OR "Perioperative" OR "Peri-operative") AND ("Hypotension" OR "Blood pressure" OR "Arterial pressure" OR "MAP" OR "Mean arterial pressure") AND ("Individuali*" OR "Personali*" OR "Optimis*" OR "Optimiz*" OR "Tailor*") AND ("Randomized controlled trial" OR "Randomized" OR "Randomised" OR "Placebo" OR "Trial" OR "RCT") | English | 19 Jan 2026 | 33 |

| **Study_ID** | | | | **Bergholz 2025** | **Futier 2017** | **Langer 2019** | **Lu 2026** | **Nicklas 2020** | **Nicklas 2024** | **Pang 2025** | **Saugel 2025** | **Zhao 2025** |
| --- | --- | --- | --- | --- | --- | --- | --- | --- | --- | --- | --- | --- |
| **Sample Size** | | | Intervention | 53 | 147 | 50 | 110 | 94 | 166 | 89 | 567 | 738 |
|  |  |  | Control | 45 | 145 | 51 | 110 | 94 | 162 | 90 | 567 | 739 |
| **Age, years** | | **Intervention** | Mean | 69 | 69.7 | 80 | 71.37 | 63 | 66 | 69 | 66 | 68.2 |
|  |  |  | SD | 7.8 | 7.1 | 4 | 6.33 | 14 | 10.4 | 6 | 4 | 8.2 |
|  |  | **Control** | Mean | 69 | 70 | 80 | 70.52 | 63 | 66 | 68 | 66 | 68.9 |
|  |  |  | SD | 8.1 | 7.5 | 4 | 7.03 | 14 | 9.6 | 5 | 4 | 8.2 |
| **Gender, Male** | | **Intervention** | N | 21 | 125 | 24 | 66 | 54 | 100 | 58 | 381 | 535 |
|  |  |  | % | 40 | 85 | 48 | 60 | 57.4 | 60 | 65.2 | 66.7 | 72.5 |
|  |  | **Control** | N | 23 | 123 | 29 | 72 | 60 | 106 | 57 | 366 | 551 |
|  |  |  | % | 51 | 85 | 57 | 65.4 | 63.8 | 65 | 63.3 | 64.6 | 74.6 |
| **BMI** | | **Intervention** | Mean | 75 | NR | 24.8 | 23.26 | 25.3 | 26.4 | 22 | 26.5 | 23.1 |
|  |  |  | SD | 11.1 | NR | 4.5 | 3.2 | 6.9 | 5.8 | 3 | 3.3 | 3.7 |
|  |  | **Control** | Mean | 80 | NR | 24.7 | 22.6 | 25.5 | 26.1 | 23 | 26.3 | 22.6 |
|  |  |  | SD | 10 | NR | 3.2 | 2.73 | 6.2 | 6.4 | 3 | 3.3 | 3.3 |
| **MAP, mmHg** | | **Intervention** | Mean | 86 | NR | NR | NR | 94 | 91 | 95 | 84.5 | 94 |
|  |  |  | SD | 6.7 | NR | NR | NR | 10 | 6 | 10 | 7.5 | 10 |
|  |  | **Control** | Mean | 89 | NR | NR | NR | 94 | 90 | 94 | 82.5 | 94 |
|  |  |  | SD | 4.1 | NR | NR | NR | 10 | 6 | 10 | 6.5 | 10 |
| **ASA** | **Physical status 2** | **Intervention** | N | 22 | 62 | NR | NR | 69 | NR | 37 | 235 | 319 |
|  |  |  | % | 42 | 42.2 | NR | NR | 11.7 | NR | 41.6 | 41.2 | 43.3 |
|  |  | **Control** | N | 23 | 54 | NR | NR | 14 | NR | 44 | 235 | 285 |
|  |  |  | % | 51 | 37.2 | NR | NR | 14.9 | NR | 48.9 | 41.4 | 38.6 |
|  | **Physical status 3** | **Intervention** | N | 27 | 84 | 31 | NR | 69 | NR | 52 | 316 | 408 |
|  |  |  | % | 51 | 57.1 | 62 | NR | 73.4 | NR | 58.4 | 55.3 | 55.4 |
|  |  | **Control** | N | 23 | 89 | 33 | NR | 67 | NR | 46 | 316 | 441 |
|  |  |  | % | 52 | 61.4 | 65 | NR | 71.3 | NR | 51.1 | 55.7 | 1.8 |
|  | **Physical status 4** | **Intervention** | N | 4 | 1 | 19 | NR | 14 | NR | 0 | 4 | 9 |
|  |  |  | % | 8 | 0.7 | 38 | NR | 14.9 | NR | 0 | 0.7 | 1.2 |
|  |  | **Control** | N | 1 | 2 | 17 | NR | 11 | NR | 0 | 7 | 13 |
|  |  |  | % | 2 | 1.4 | 33 | NR | 11.7 | NR | 0 | 1.2 | 1.8 |
| **HTN Medications** | **Any** | **Intervention** | N | 45 | 100 | 0 | 110 | NR | NR | NR | 327 | NR |
|  |  |  | % | 85 | 68 | 0 | 100 | NR | NR | NR | 57.3 | NR |
|  |  | **Control** | N | 38 | 97 | 1 | 110 | NR | NR | NR | 296 | NR |
|  |  |  | % | 84 | 66.9 | 2 | 100 | NR | NR | NR | 52.2 | NR |
|  | **β-Blocker** | **Intervention** | N | 16 | NR | NR | NR | NR | NR | NR | 172 | 78 |
|  |  |  | % | 30 | NR | NR | NR | NR | NR | NR | 30.1 | 10.6 |
|  |  | **Control** | N | 7 | NR | NR | NR | NR | NR | NR | 138 | 92 |
|  |  |  | % | 16 | NR | NR | NR | NR | NR | NR | 24.3 | 12.4 |
|  | **ACEI** | **Intervention** | N | 13 | 71 | NR | NR | NR | NR | 6 | 117 | 132 |
|  |  |  | % | 25 | 48.2 | NR | NR | NR | NR | 6.7 | 20.5 | 17.9 |
|  |  | **Control** | N | 3 | 72 | NR | NR | NR | NR | 7 | 101 | 152 |
|  |  |  | % | 7 | 49.6 | NR | NR | NR | NR | 7.8 | 17.8 | 20.6 |
|  | **ARBs** | **Intervention** | N | 11 | 71 | NR | NR | NR | NR | 6 | 131 | 132 |
|  |  |  | % | 21 | 48.2 | NR | NR | NR | NR | 6.7 | 22.9 | 17.9 |
|  |  | **Control** | N | 11 | 72 | NR | NR | NR | NR | 5 | 112 | 152 |
|  |  |  | % | 24 | 49.6 | NR | NR | NR | NR | 5.6 | 19.8 | 20.6 |
|  | **CCBs** | **Intervention** | N | 5 | NR | NR | NR | NR | NR | NR | 126 | 265 |
|  |  |  | % | 9 | NR | NR | NR | NR | NR | NR | 22.1 | 36 |
|  |  | **Control** | N | 7 | NR | NR | NR | NR | NR | NR | 89 | 271 |
|  |  |  | % | 16 | NR | NR | NR | NR | NR | NR | 15.7 | 36.7 |
|  | **Diuretics** | **Intervention** | N | 10 | 24 | NR | NR | NR | NR | NR | 111 | 31 |
|  |  |  | % | 19 | 16.3 | NR | NR | NR | NR | NR | 19.4 | 4.2 |
|  |  | **Control** | N | 7 | 20 | NR | NR | NR | NR | NR | 87 | 30 |
|  |  |  | % | 16 | 13.8 | NR | NR | NR | NR | NR | 15.3 | 4.1 |
| **Risk Factors** | **DM** | **Intervention** | N | 6 | 77 | NR | NR | NR | NR | 12 | 105 | 210 |
|  |  |  | % | 11 | 52.4 | NR | NR | NR | NR | 13.5 | 18.4 | 28.5 |
|  |  | **Control** | N | 10 | 73 | NR | NR | NR | NR | 8 | 86 | 222 |
|  |  |  | % | 22 | 50.3 | NR | NR | NR | NR | 8.9 | 15.2 | 30 |
|  | **Renal impairment** | **Intervention** | N | 4 | 20 | 6 | NR | 22 | NR | 0 | 396 | 35 |
|  |  |  | % | 8 | 13.7 | 12 | NR | 23.4 | NR | 0 | 69.4 | 4.8 |
|  |  | **Control** | N | 1 | 17 | 11 | NR | 25 | NR | 0 | 377 | 32 |
|  |  |  | % | 2 | 11.9 | 22 | NR | 26.6 | NR | 0 | 66.5 | 4.3 |
|  | **Expected duration of surgery > 2h** | **Intervention** | N | NR | 147 | 18 | 110 | NR | NR | 89 | 448 | 738 |
|  |  |  | % | NR | 100 | 36 | 100 | NR | NR | 100 | 78.5 | 100 |
|  |  | **Control** | N | NR | 145 | 22 | 110 | NR | NR | 90 | 456 | 739 |
|  |  |  | % | NR | 100 | 43 | 100 | NR | NR | 100 | 80.4 | 100 |
|  | **Smoking** | **Intervention** | N | NR | NR | NR | NR | NR | NR | 46 | 133 | 267 |
|  |  |  | % | NR | NR | NR | NR | NR | NR | 51.7 | 23.3 | 36.5 |
|  |  | **Control** | N | NR | NR | NR | NR | NR | NR | 40 | 142 | 255 |
|  |  |  | % | NR | NR | NR | NR | NR | NR | 44.4 | 25 | 34.8 |
|  | **CAD** | **Intervention** | N | 4 | 20 | NR | NR | NR | NR | 5 | 71 | 124 |
|  |  |  | % | 8 | 13.6 | NR | NR | NR | NR | 5.6 | 12.4 | 16.8 |
|  |  | **Control** | N | 5 | 32 | NR | NR | NR | NR | 4 | 67 | 134 |
|  |  |  | % | 11 | 22.1 | NR | NR | NR | NR | 4.4 | 11.8 | 18.1 |
|  | **HF** | **Intervention** | N | 3 | 26 | NR | NR | NR | NR | 6 | 21 | 13 |
|  |  |  | % | 8 | 17.7 | NR | NR | NR | NR | 6.7 | 3.7 | 1.8 |
|  |  | **Control** | N | 1 | 38 | NR | NR | NR | NR | 6 | 17 | 8 |
|  |  |  | % | 2 | 26.2 | NR | NR | NR | NR | 6.7 | 3 | 1.1 |
|  | **Cancer surgery** | **Intervention** | N | 44 | NR | 28 | 110 | NR | NR | 89 | 430 | NR |
|  |  |  | % | 83 | NR | 56 | 100 | NR | NR | 100 | 75.3 | NR |
|  |  | **Control** | N | 37 | NR | 34 | 110 | NR | NR | 90 | 427 | NR |
|  |  |  | % | 82 | NR | 67 | 100 | NR | NR | 100 | 75.3 | NR |
| **Surgery type** | **Abdominal** | **Intervention** | N | 32 | 138 | 30 | 110 | 94 | 166 | 89 | 571 | 738 |
|  |  |  | % | 60 | 93.9 | 60 | 100 | 100 | 100 | 100 | 100 | 100 |
|  |  | **Control** | N | 28 | 140 | 27 | 110 | 94 | 162 | 90 | 567 | 739 |
|  |  |  | % | 62 | 96.6 | 53 | 100 | 100 | 100 | 100 | 100 | 100 |
|  | **Other** | **Intervention** | N | 21 | 9 | 20 | 0 | 0 | 0 | 0 | 0 | 0 |
|  |  |  | % | 40 | 6.1 | 40 | 0 | 0 | 0 | 0 | 0 | 0 |
|  |  | **Control** | N | 17 | 5 | 24 | 0 | 0 | 0 | 0 | 0 | 0 |
|  |  |  | % | 38 | 3.4 | 47 | 0 | 0 | 0 | 0 | 0 | 0 |
| N; number of patients, SD; standard deviation, BMI; body mass index, MAP; mean arterial pressure, ASA; American Society of Anesthesiologists Physical Status Classification System, HTN; hypertension, ACEI; Angiotensin-Converting Enzyme Inhibitor, ARB; Angiotensin II Receptor Blocker, CCBs;Calcium channel blockers, DM; diabetes mellitus, CAD; coronary artery disease, HF; heart failure | | | | | | | | | | | | |

**Supplementary Table 2.** Baseline characteristics of the included patients.

**Supplementary Figure S1.** Random-effect model of MI rates.

**Supplementary Figure S2.** Random-effect model of delirium rates.

**Supplementary Figure S3.** Random-effect model of postoperative cognitive dysfunction rates.

**Supplementary Figure S4.** Random-effect model of postoperative composite complications rates.

**Supplementary Figure S5.** Random-effect model of length of hospital stay.

**Supplementary Figure S6.** Leave-one-out sensitivity analysis of mean intraoperative MAP.

**Supplementary Figure S7.** Leave-one-out sensitivity analysis of length of hospital stay.
